# Study on the Interaction Mechanism Between Linoleic Acid, Myristic Acid and Rice Protein by Spectroscopy

**DOI:** 10.3390/foods15152604

**Published:** 2026-07-25

**Authors:** Ligen Wu, Mouchang Fang, Anna Wang

**Affiliations:** 1 College of Food Science and Engineering, Henan University of Technology, Zhengzhou 450001, China; mouchang2026@163.com; 2 National Engineering Research Center of Wheat and Corn Further Processing, Henan University of Technology, Zhengzhou 450001, China

**Keywords:** rice protein, fatty acids, fluorescence spectrum, UV spectrum

## Abstract

Myristic acid (MA) and linoleic acid (LA) have been widely utilized as food additives in the food industry, and have also demonstrated notable pharmacological activities. However, the molecular mechanisms underlying their interactions with rice protein (RP) remain poorly understood and warrant further systematic investigation. A comprehensive characterization of the molecular interactions between myristic acid, linoleic acid, and rice protein was conducted through UV absorption, fluorescence emission, and molecular docking analyses, enabling the determination of fluorescence quenching pathways, binding affinities, and the predominant forces responsible for complex formation. The results indicate that both myristic acid and linoleic acid induce the static quenching of rice protein fluorescence. The interaction between these fatty acids and rice protein is primarily governed by electrostatic forces. Synchronous fluorescence spectroscopy reveals that the addition of myristic acid causes negligible alterations in tyrosine fluorescence intensity, while significantly reducing tryptophan fluorescence. In contrast, linoleic acid leads to a marked decrease in fluorescence intensities of both tyrosine and tryptophan residues in rice protein. Three-dimensional fluorescence spectroscopy revealed a consistent decrease in the fluorescence intensity of rice protein upon the addition of both myristic acid and linoleic acid. Molecular docking simulations further indicated that linoleic acid exhibits a higher binding affinity toward rice protein, as reflected by its more favorable binding energy. This study elucidates the interaction mechanisms between rice protein and myristic acid or linoleic acid, providing a theoretical foundation for the practical development of rice protein-based products.

## 1. Introduction

Proteins constitute an indispensable component of the human diet, playing a pivotal role in growth, tissue regeneration, and immune system function [1,2]. Protein is the second most prevalent type of storage substance in rice seeds, generally accounting for about 8–10% of the dry weight of brown rice [3]. Rice protein (RP), as a plant-based protein source, exhibits a balanced amino acid profile [4], low allergenicity [5], high digestibility [6], and negligible cholesterol content [7]. Moreover, rice protein has been shown to exhibit a range of beneficial biological activities, including antioxidant [8], anti-inflammatory [9], anti-obesity [10], and antihypertensive [11] properties, as supported by emerging in vitro and in vivo studies. Rice protein has been shown to interact with bioactive compounds, such as gallic acid [12]. This further underscores its significance as a key constituent in the development of personalized nutrition strategies and functional food formulations [13]. Consequently, RP has attracted increasing attention from both researchers and the food industry.

Fatty acids, classified as saturated and unsaturated, play crucial biological roles, including energy storage, participation in biomembrane formation, and serving as signaling molecules that regulate physiological processes [14]. Myristic acid (MA), also known as tetradecanoic acid, is a saturated fatty acid commonly utilized in the food industry for the synthesis of flavor compounds and as a flavor enhancer. Myristic acid has many advantages, such as low cost and low toxicity [15].

Linoleic acid (LA), an essential polyunsaturated fatty acid (PUFA) for human nutrition, is abundantly present in plant-derived oils such as cottonseed, sunflower, and peanut oils, while exhibiting low concentrations in animal fats [16]. Emerging evidence suggests that LA possesses potential anti-carcinogenic [17] and anti-inflammatory properties [18]. Moreover, investigating small molecule–protein interactions is of considerable pharmacokinetic and pharmacodynamic relevance in the context of therapeutic interventions for diabetes, breast cancer, bladder cancer, and other malignancies [19,20].

Relevant studies have shown that fatty acids can interact with proteins under certain conditions, thereby influencing proteins’ structural and functional properties [21]. Under normal circumstances, the combination of fatty acids with proteins will cause changes to the secondary and tertiary structures of the proteins. In addition, fatty acids have an impact on the emulsifying property [22], foaming property [23], gelling property [24] and solubility of proteins [25].

In current research on fatty acid–protein interactions, Xu et al. [26] investigated the interaction between rice bran protein and conjugated linoleic acid, reporting a concentration-dependent quenching of the protein’s intrinsic fluorescence, indicative of molecular association. Thermodynamic analysis revealed that the binding process is spontaneous. Conjugated linoleic acid can be taken together with rice gluten as a dietary supplement for fat loss in the fitness industry, which may provide theoretical guidance for improving the performance of some existing nutritional products in the food industry. In a comparative study, Hui Yang [27] investigated the binding mechanisms and intermolecular interactions between β-lactoglobulin and six fatty acids of varying chain lengths. The fatty acid set included four saturated species with chain lengths from C12:0 to C18:0 and two unsaturated C18 fatty acids (C18:1 and C18:2). Fluorescence quenching data have indicated a static quenching mechanism for all interactions, primarily driven by hydrophobic forces. Binding affinity increased with the length of the fatty acid hydrocarbon chain. Furthermore, the binding of fatty acids disrupted the microenvironments of tyrosine and tryptophan residues in β-LG, leading to conformational changes that altered the protein’s secondary structure. This will help us better understand the interactions of nutrients in food.

Although ligand–protein interactions have been widely explored, mechanistic insights into the binding of myristic acid and linoleic acid to rice protein are still scarce. This investigation employs MA and LA as model ligands to systematically examine their concentration-dependent interactions with RP. The primary objectives are to elucidate the molecular basis underlying MA and LA–RP binding dynamics, and to characterize ligand-induced conformational alterations in RP tertiary structure. Research shows that RP represents a promising plant-based alternative to animal-derived proteins (e.g., casein and whey) in sports nutrition formulations [28]. The investigation into rice protein–fatty acid complexes holds significant reference value for the development of protein components in specialized nutritional products, including functional nutrient powders, sports supplements, clinical enteral formulas, and infant formula. The principal developmental advantages of such complexes lie in their capacity for synergistic nutrient fortification, enabling the concurrent supplementation of high-quality protein and essential fatty acids within a single matrix. Furthermore, the low-allergenic profile of native rice proteins renders them particularly suitable for consumption by sensitized populations. The mechanistic insights derived from fatty acid–rice protein interaction studies provide a theoretical framework for optimizing the functional performance of existing nutritional products in the food industry.

## 2. Materials and Methods

### 2.1. Experimental Materials

Rice protein (purity > 80%): Shanxi Mengtai Biological Products Co., Ltd. (Xi’an, China). Myristic acid (purity > 97%) and linoleic acid (purity > 95%): Shanghai McLean Biochemical Technology Co., Ltd. (Shanghai, China). Na_2_HPO_4_ and NaH_2_PO_4_ (purity ≥ 99%): Tianjin Kemiou Chemical Reagent Co., Ltd. (Tianjin, China).

### 2.2. Instruments and Equipment

DF-101S Collecting-Type Constant-Temperature Magnetic Stirrer: Henan Baize Instrument Co., Ltd. (Zhengzhou, China). F98 Fluorescence Spectrophotometer, UV7600 Dual-Beam UV-Vis Spectrophotometer: Shanghai Lengguang Technology Co., Ltd. (Shanghai, China).

### 2.3. Experimental Methods

RP was solubilized in 0.01 M PBS (pH 7.0) under continuous magnetic stirring to achieve dissolution [26]. The final concentration of the RP solution was standardized at 0.4 mg/mL. MA and LA were dissolved in 100 mL of ethanol solution to prepare a 0.2 mg/mL fatty acid solution. These solutions were then diluted with 0.01 mol/L PBS (pH 7.0) to final concentrations of 0, 0.025, 0.05, 0.075, 0.1, and 0.125 mg/mL, respectively. The resulting solutions were stored protected from light at –4 °C for later use.

Solutions of fatty acids at different concentrations were mixed with protein solution in a 1:1 volume ratio. The mixtures reacted at 298 K and 310 K for 20 min before use. Mixed solutions with fatty acid concentrations of 0, 0.0125, 0.025, 0.0375, 0.05 and 0.0625 mg/mL were obtained for subsequent use.

### 2.4. UV Spectroscopy

UV-Vis spectroscopic measurements were performed employing a UV7600 double-beam spectrophotometer (Lengguang Technology Co., Ltd., Shanghai, China). The absorption spectra of MA–RP and LA–RP were recorded across the wavelength range of 200 to 400 nm.

### 2.5. Fluorescence Spectroscopy

The fluorescence spectral characterization of MA–RP (myristic acid–rice protein) and LA–RP (linoleic acid–rice protein) was conducted with an F98 Fluorescence Spectrophotometer (Lengguang Technology Co., Ltd., Shanghai, China). For intrinsic fluorescence measurements, the excitation wavelength was set at 280 nm, and the emission spectra were recorded over the wavelength range of 300 to 500 nm. The measured samples were preheated at 298 K and 310 K.

### 2.6. Determination of Synchronous Fluorescence Spectra

Synchronous fluorescence measurements were conducted at room temperature. The critical spectral parameters were set as predefined wavelength gaps (Δλ = 15 nm and 60 nm) between the excitation and emission monochromators, and the synchronous scanning spectrum was recorded across the 200–500 nm region.

### 2.7. Determination of Three-Dimensional Fluorescence Spectra

The three-dimensional fluorescence spectrum of the sample was recorded at room temperature. The recording range was set from 200 to 600 nm with incremental steps of 10 nm, and the scanning rate was 10,000 nm/min.

### 2.8. Molecular Docking Simulations

For molecular docking validation, the molecular structure of the primary active ingredient was downloaded from the PubChem database and saved in SDF format. The three-dimensional crystal structure of the target protein was downloaded from the PDB database and saved in PDB format. Using PyMOL software, water molecules and ligands were removed from the protein structure, which was then saved in PDB format. The Getbox Plugin was employed to obtain the docking pocket parameters. The processed protein and active ingredient files were imported into AutoDock Tools 1.5.6 and converted to PDBQT format. Molecular docking was then performed using AutoDock Vina 1.1.2. Finally, the docking results were visualized using PyMOL 2.6.0 software.

### 2.9. Statistical Analyses

Origin 2024 was used to make the graph, and Pymol was used for the 3D mapping of the molecular docking.

## 3. Results and Discussion

### 3.1. Ultraviolet Spectra

UV-Vis absorption spectroscopy is a widely employed technique for investigating interactions between proteins and small molecules [29]. The absorption peak near 210 nm arises from the peptide backbone, whereas the band at approximately 280 nm is attributed to π→π* transitions of aromatic amino acids [30]. This technique is commonly employed to confirm protein structural changes and complex formation.

**Figure 1:**
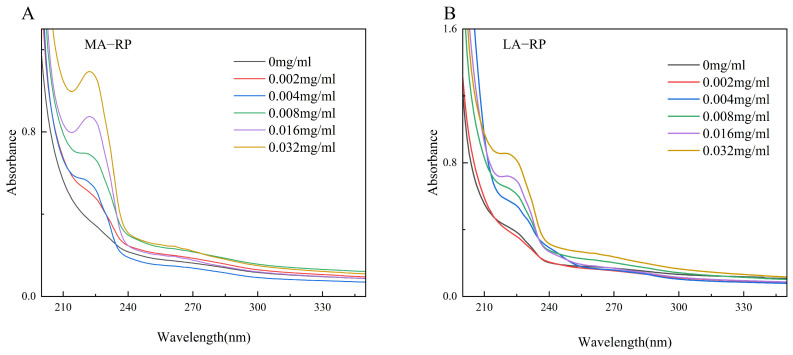
UV-Vis spectra of myristic acid (MA), linoleic acid (LA) and rice protein (RP). (**A**) shows the UV-Vis spectrum of the MA–RP system, and (**B**) shows the UV-Vis spectrum of the LA–RP system.

Figure 1 presents the UV-Vis spectra of RP in the presence of varying concentrations of the two fatty acids. The UV absorption spectra of the composite systems exhibit a characteristic peak near 220 nm, which reflects the structural framework of RP. In the MA–RP system, an increase in MA concentration leads to a red shift of the RP absorption peak to 222 nm. In contrast, in the LA–RP system, the peak at 220 nm undergoes notable changes with increasing LA concentration, yet no significant red or blue shift is observed. These findings indicate that fatty acids form complexes with rice protein, leading to conformational changes in the protein [30]. The UV-Vis spectrum did not exhibit a distinct peak at approximately 280 nm, and only a relatively weak absorption band was observed, reflecting the interaction of fatty acids with protein. The observed peak is predominantly ascribed to electronic transitions (π→π*) of aromatic amino acid residues, including tryptophan, tyrosine, and phenylalanine, embedded in the protein structure. The spectral changes indicate that fatty acids interact with RP binding to its internal cavities, inducing a less polar microenvironment around aromatic residues. The results suggest that fatty acid-induced oxidative modification triggers structural perturbations in protein, resulting in changes to its spatial conformation [31]. The interaction between fatty acids and RP can be further probed using fluorescence spectroscopy.

### 3.2. Fluorescence Quenching Mechanism

Fluorescence spectroscopy was employed to systematically investigate the interaction mechanisms between RP and varying concentrations of myristic acid and linoleic acid. The binding constant (K_a_), number of binding sites (n) and predominant interaction forces between fatty acids and rice protein were quantitatively determined by monitoring the quenching kinetics, maximum emission wavelength shifts and synchronous fluorescence spectral changes of the intrinsic fluorescence (primarily from tryptophan residues) of rice protein. Fluorescence spectroscopy is fundamentally a technique for probing changes in the local microenvironment of fluorophores, where structural modifications within the fluorophore’s vicinity can influence the fluorescence signal [31].

**Figure 2:**
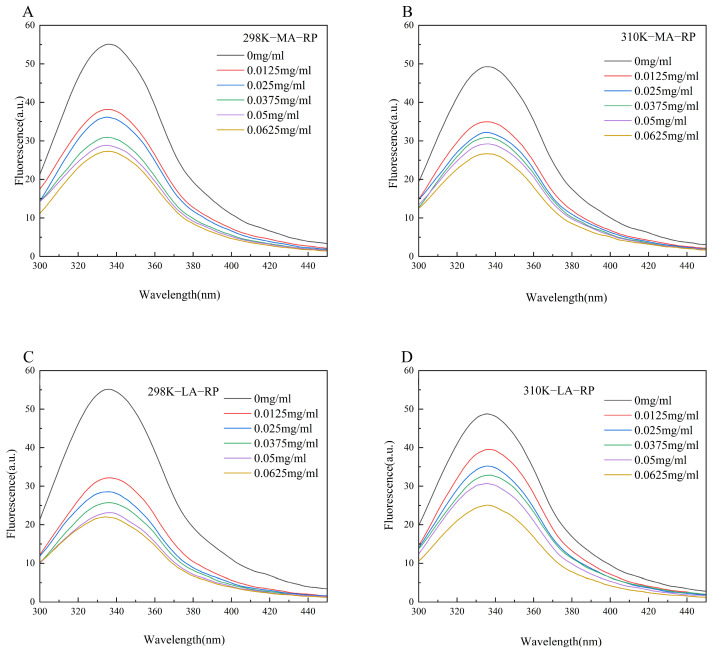
The fluorescence quenching spectra of the interaction between myristic acid (MA) and linoleic acid (LA) with rice protein (RP) at 298 K (**A**,**C**) and 310 K (**B**,**D**) were studied.

The effects of two fatty acids on the intrinsic fluorescence of RP were measured at 298 K and 310 K. As shown in Figure 2, in the MA–RP system, the fluorescence emission peak is centered at approximately 340 nm with a maximum intensity of 55.09 a.u., whereas in the LA–RP system, the peak appears at around 330 nm with a maximum intensity of 55.14 a.u. Compared to the control, the characteristic fluorescence peak of RP at ~335 nm under excitation at 280 nm exhibited a significant dose-dependent decrease with increasing fatty acid concentration. This behavior is consistent with fluorescence quenching, indicating an effective interaction between fatty acid molecules and the fluorescent chromophores (primarily tryptophan residues) within rice protein. The observed fluorescence quenching is likely attributed to the association of fatty acids with hydrophobic pockets or defined binding sites on RP, resulting in direct interactions with tryptophan and tyrosine residues and the subsequent formation of non-fluorescent adducts, which diminishes the effective population of emissive chromophores.

#### 3.2.1. Quenching Type

The Stern–Volmer equation was employed to calculate the quenching type between fatty acids and RP.

F_0_/F = K_q_τ_0_[Q] + 1 = K_sv_[Q] + 1 (1)

F_0_ denotes intrinsic fluorescence without added fatty acids; F represents intrinsic fluorescence with varying fatty acid concentrations; K_q_ denotes quenching rate; τ_0_ denotes white fluorescence lifetime, equal to10^−8^ s; K_sv_ denotes Stern–Volmer quenching constant [32].

**Figure 3:**
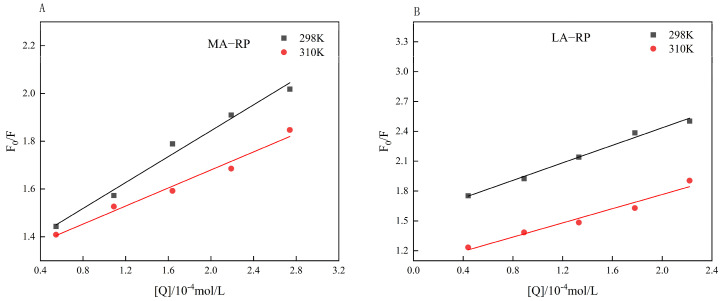
Stern–Volmer equations fitting diagram of the interaction between myristic acid (MA), linoleic acid (LA) and rice protein (RP). (**A**) and (**B**) show the fitted plots of the SV equation for the MA-RP and LA-RP systems, respectively.

Figure 3 displays the Stern–Volmer equation fitting plots of the interactions between MA, LA, and RP. The fitting of the fluorescence quenching data at 298 K and 310 K using the Stern–Volmer equation revealed excellent linearity (R^2^ > 0.95) for both MA–RP and LA–RP systems across the experimental concentration range, suggesting that the quenching process may predominantly follow a single dynamic collision or static binding mechanism. To further elucidate the quenching mechanism, the corresponding Stern–Volmer quenching constants (K_sv_) were calculated. Table 1 shows the Stern–Volmer equation and the coefficient of determination for the interaction between MA, LA, and RP. The results show that K_sv_ values for both MA–RP and LA–RP systems decreased significantly with increasing temperature from 298 K to 310 K, a trend consistent with static quenching. This finding provides critical thermodynamic evidence for understanding the nature of interactions between fatty acids and rice protein. Moreover, the K_q_ values for both MA–RP and LA–RP systems exceeded the maximum diffusion-controlled quenching constant of 2 × 10^10^ L/mol^−1^ s^−1^, indicating static quenching. Under identical temperatures, the K_sv_ and K_q_ values for the LA–RP system were consistently higher than those for the MA–RP system, suggesting a stronger affinity between LA and RP.

#### 3.2.2. Binding Site Association Constant

The binding constant and binding sites of fatty acids with RP were calculated via a double-logarithmic equation.

lg[(F_0_ − F)/F ] = lgK_a_ + nlg[Q] (2)

where F_0_ denotes intrinsic fluorescence without added fatty acids; F represents intrinsic fluorescence with varying fatty acid concentrations; K_a_ denotes the binding constant; n denotes the number of binding sites; [Q] is the quencher concentration.

The association constant (K_a_) serves as a quantitative indicator of the affinity between a protein and its ligand. K_a_ values can be employed to reflect the binding strength of interactions between MA, LA, and RP, with higher K_a_ values indicating stronger binding interactions.

**Figure 4:**
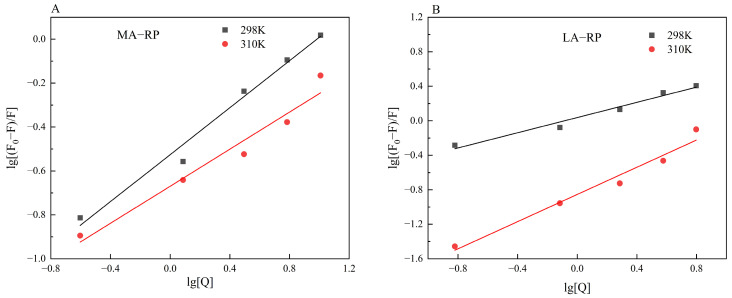
Double logarithmic equations fitting diagram of the interaction between myristic acid (MA), linoleic acid (LA) and rice protein (RP). (**A**) and (**B**) show double logarithmic equations fitting diagram for the MA-RP and LA-RP systems, respectively.

As shown in Table 2 and Figure 4, among rice protein–fatty acid complexes, the K_a_ values for both MA–RP and LA–RP systems decreased with increasing temperature, indicating that these interactions are exothermic in nature. The LA–RP system exhibited the highest association constant (K_a_), at 298 K. These results indicate a stronger binding strength. The number of binding sites for both fatty acids were determined to be around 0.5, significantly below the expected value of 1. This observation implies that the initial binding of fatty acids may trigger conformational rearrangements of the protein, leading to a negative cooperative effect on subsequent binding events.

#### 3.2.3. Interaction Force Type

Thermodynamic parameters (enthalpy change, entropy change, and Gibbs free energy) were calculated using the Van’t Hoff equation and thermodynamic equations.

(3) lnK=−ΔH/RT+ΔS/R

(4) ΔG=ΔH−TΔS

K denotes the binding constant; T denotes absolute temperature; R denotes the gas constant, equal to 8.314 J·mol^−1^·K^−1^; ΔH denotes enthalpy change; ΔS denotes entropy change; ΔG denotes the change in Gibbs free energy.

Non-covalent interactions are commonly involved in the formation of complexes between proteins and bioactive small molecules [33]. Non-covalent binding between ligands and proteins typically involves four types of interactions, namely, hydrophobic interactions, van der Waals forces, hydrogen bonding, and electrostatic interactions. These interactions can be differentiated through thermodynamic analysis based on enthalpy change (ΔH) and entropy change (ΔS). Specifically, hydrophobic interactions are characterized by ΔH > 0 and ΔS > 0; van der Waals forces or hydrogen bonds are associated with ΔH < 0 and ΔS < 0, while electrostatic interactions are indicated by ΔH < 0 and ΔS > 0.

As presented in Table 3, electrostatic interactions serve as the predominant driving force for the binding of both MA and LA to RP. Moreover, the negative ΔG values obtained for all binding reactions indicate that the complex formation processes were thermodynamically spontaneous. The absolute values of ΔG for both systems increased with rising temperature, indicating that the electrostatic interactions are highly temperature-sensitive, and that elevated temperatures may weaken the binding affinity of these systems. Furthermore, the negative ΔH values indicate that both binding interactions are exothermic, which accounts for the observed decrease in binding constants with increasing temperature.

These findings demonstrate that RP exhibits strong binding affinity for certain unsaturated fatty acids. This implies that the protein can effectively encapsulate or sequester fatty acids, thereby mitigating their oxidative degradation during food processing and storage. Consequently, a comprehensive understanding of the binding characteristics between different fatty acids and rice protein in rice milk systems can enable food technologists to make more scientifically informed choices regarding lipid selection. Ultimately, this may offer consumers higher-quality products with expanded options.

### 3.3. Synchronous Fluorescence Spectra

Synchronous fluorescence spectroscopy, implemented by fixing the wavelength difference (Δλ) and concurrently scanning the excitation and emission monochromators, facilitates the selective resolution of fluorescence emission features associated with tryptophan and tyrosine residues. This approach is commonly applied to investigate alterations in the microenvironment of protein chromophores upon interaction with small-molecule ligands, with the distinct advantage of minimizing spectral overlap and suppressing scattering interference [34]. Shifts in absorption bands observed in synchronous spectra indicate alterations in the local microenvironment of amino acid residues, with red shifts signifying increased polarity and blue shifts reflecting enhanced hydrophobicity [35]. The use of Δλ = 15 nm and Δλ = 60 nm enables the selective enhancement of changes in the vicinity of tyrosine (Tyr) and tryptophan (Trp) residues, respectively [36]. The objective is to elucidate the conformational rearrangements and microenvironmental modifications occurring during the binding process.

**Figure 5:**
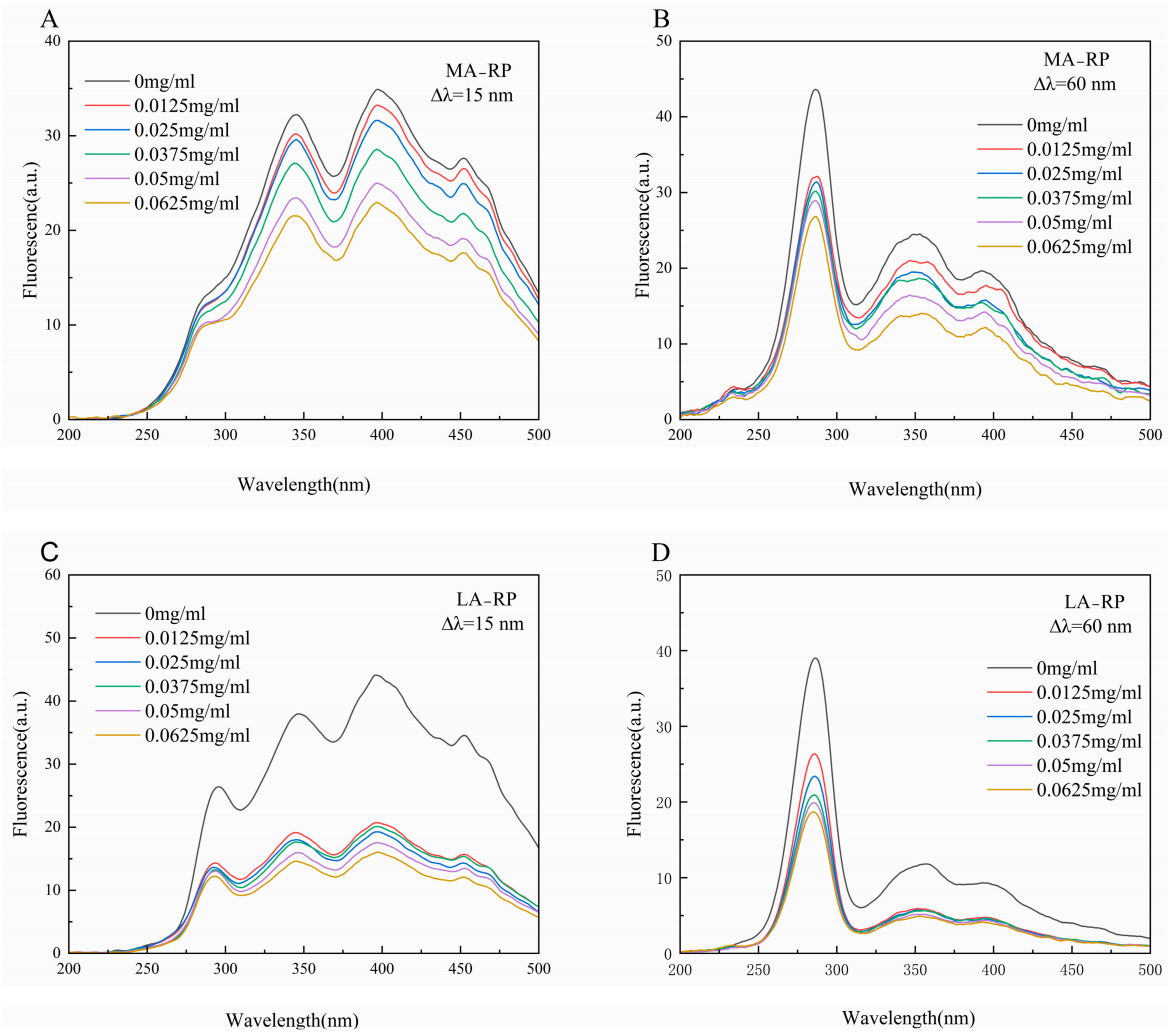
Synchronous fluorescence spectra of myristic acid (**A**,**B**) and linoleic acid (**C**,**D**) interacting with rice protein (RP).

Figure 5 presents the synchronous fluorescence spectra of MA, LA and RP complexes at 298 K under varying concentrations. The results demonstrate a significant decrease in the fluorescence intensity of RP upon the addition of fatty acids, indicating the effective quenching of its intrinsic fluorescence by the two fatty acids. Moreover, LA exhibited a greater quenching effect on RP compared to MA, which may be attributed to the differing degrees of saturation of the fatty acids. Additionally, the fluorescence intensity and quenching extent at Δλ = 60 nm (Trp) were lower than those at Δλ = 15 nm (Tyr), suggesting that the binding sites of both fatty acids on rice protein are in closer proximity to Trp residues.

### 3.4. Three-Dimensional Fluorescence Spectroscopy

Three-dimensional fluorescence spectroscopy provides a more vivid and comprehensive representation of fluorescence intensity variations as a function of excitation and emission wavelengths, offering richer information compared to conventional fluorescence spectroscopy [37].

**Figure 6:**
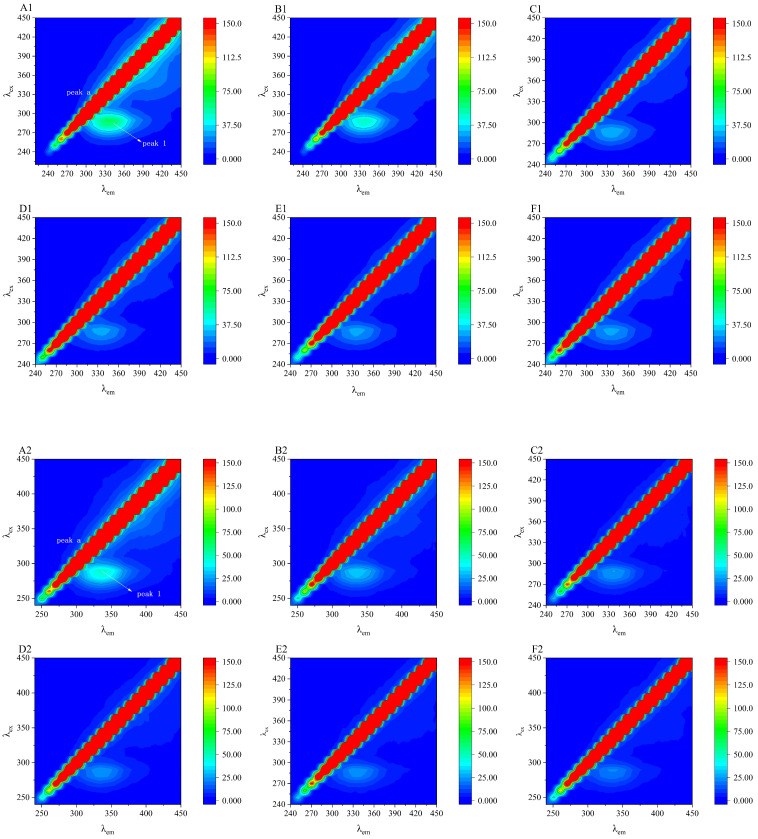
Three–dimensional fluorescence spectra of MA–RP and LA–RP interactions, where (**A1**–**F1**) and (**A2**–**F2**) represent the spectra of myristic acid (MA) and linoleic acid (LA) at concentrations of 0, 0.0125, 0.025, 0.0375, 0.05, and 0.0625 mg/mL, respectively.

Figure 6 presents the three-dimensional fluorescence spectra of RP in the presence of varying concentrations of MA and LA. In the spectra, peak a corresponds to the Rayleigh scattering peak, while peak 1 mainly reflects the fluorescence characteristics of tryptophan and/or tyrosine residues of RP. As shown in Figure 6, the fluorescence intensity of peak 1 in the composite systems decreased in a concentration-dependent manner with increasing MA and LA concentrations. This observation indicates that interactions occur between MA/LA and RP, leading to the formation of stable complexes.

Furthermore, with an increasing fatty acid concentration, no significant shift was observed for the Rayleigh scattering peak; however, the characteristic fluorescence peak of RP exhibited a slight blue shift. Specifically, the emission maximum of the MA–RP system shifted from 336 nm to 334 nm, while that of the LA–RP system shifted from 338 nm to 336 nm. These blue shifts suggest an increase in hydrophobicity and a decrease in polarity around the fluorophores, implying that the binding of fatty acids induces conformational changes in RP, accompanied by the folding and conformational rearrangement of the polypeptide chains, which in turn alters the secondary structure of the protein [38]. The observed fluorescence decrease and blue shift are attributed to changes in the polarity of the microenvironment surrounding key aromatic amino acid residues (e.g., tryptophan) upon complex formation, which in turn modulates their fluorescence emission properties. Furthermore, the trend of changes in characteristic peak intensities in the three-dimensional fluorescence spectra was consistent with the results of the fluorescence quenching titration experiments, thereby enhancing the reliability of the quantitative data.

### 3.5. Molecular Docking

**Figure 7:**
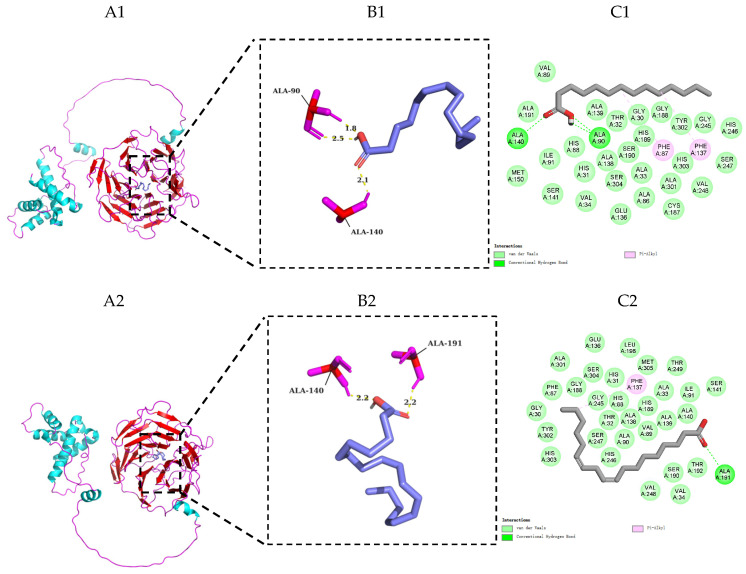
Molecular docking results of myristic acid (MA) (**A1**–**C1**) and linoleic acid (LA) (**A2**–**C2**) with rice glutelin, respectively. (**A1**,**A2**) represent three-dimensional plots, (**B1**,**B2**) represent magnified views, and (**C1**,**C2**) represent two-dimensional plots. In panels (**B1**,**B2**), purple represents fatty acids, pink represents rice glutelin, and yellow represents hydrogen bonds.

Molecular docking was utilized to explore the interaction between fatty acids and rice glutelin, with myristic acid–rice glutelin and linoleic acid–rice glutelin serving as representative complexes. Figure 7 presents the most stable binding conformations of the complexes. As supported by docking studies, lower binding energy values correspond to higher binding probabilities and stronger intermolecular interactions [39]. The binding energies of myristic acid–rice glutelin and linoleic acid–rice glutelin were calculated as −5.4 kcal/mol and −5.6 kcal/mol, respectively. Both values are negative, indicating a favorable binding affinity between rice glutelin and fatty acids.

The three-dimensional and two-dimensional docking models clearly demonstrate the embedding of fatty acids into a cavity within rice glutelin, accompanied by interactions with specific amino acid residues. The 2D model reveals that the carboxyl terminus of MA forms two conventional hydrogen bonds with ALA90 and ALA140 of rice glutelin, with bond lengths of 1.8 Å and 2.1–2.5 Å, respectively. Additionally, the saturated long carbon chain of MA engages in van der Waals interactions with residues in the binding pocket, including ALA33, ALA86, ALA138, ALA139, ALA191, ALA301; GLY30, GLY188, GLY245; SER141, SER190, SER247, SER304; ILE91, et al. Furthermore, π-alkyl interactions are observed with PHE87 and PHE137 residues.

The carboxyl terminus of LA forms two hydrogen bonds with ALA191 of rice glutelin, both with a bond length of 2.2 Å. The unsaturated long carbon chain of LA (containing two double bonds) engages in extensive van der Waals interactions with multiple residues in the binding pocket, including ALA33, ALA90, ALA138, ALA139, ALA140, ALA301; VAL34, VAL89, VAL248; GLY30, GLY188, GLY245; SER141, SER190, SER247, SER304; HIS31, HIS88, HIS189, HIS246, HIS303; MET305; LEU198, ILE91, PHE87, et al. Additionally, the carbon chain participates in π-alkyl interactions with residues such as PHE137. These interactions play a critical role in stabilizing the three-dimensional structure of the protein, thereby enhancing binding stability.

## 4. Conclusions

Fatty acids and RP represent indispensable components in modern food industry applications. Fatty acids primarily contribute to energy provision and fundamental health support, whereas RP has gained prominence in premium nutrition and specialized diets due to its safety, mild properties, and high digestibility. The interaction mechanism between fatty acids and RP was investigated using spectroscopic methods. The strategic conjugation of RP with fatty acids significantly broadens its application spectrum in functional food systems and nutritional additive formulations. UV-Vis spectroscopy revealed a gradual increase in absorbance with increasing fatty acid concentration. With increasing fatty acid concentration, the intrinsic fluorescence of rice protein progressively diminished. Both LA and MA exhibited the static quenching of RP fluorescence, and the interactions were spontaneous exothermic processes, primarily driven by electrostatic interactions. Molecular docking provides molecular-level insights into the interaction mechanisms between the two fatty acids and rice protein. The findings provide a basis for developing food products with enhanced nutritional benefits. The stable binding between rice protein and fatty acids can improve the bioaccessibility of protein, facilitating the design of tailored nutritional foods better suited for infants, the elderly, or individuals with digestive sensitivities.

## Figures and Tables

**Table 1:** Stern–Volmer equation and coefficient of determination for the interaction of myristic acid (MA) and linoleic acid (LA) with rice protein (RP), respectively.

Sample	T/K	Stern–Volmer Equation	*K_sv_* (10^4^ L mol^−1^)	*K_q_* (10^12^ L mol^−1^ s^−1^)	*R* ^2^
MA–RP	298 K	F_0_/F = 0.2712[Q] + 1.3017	0.2712	0.2712	0.9850
	310 K	F_0_/F = 0.1890[Q] + 1.3021	0.1890	0.1890	0.9779
LA–RP	298 K	F_0/_F = 0.4402[Q] + 1.5553	0.4402	0.4402	0.9910
	310 K	F_0/_F = 0.3569[Q] + 1.0518	0.3569	0.3569	0.9631

**Table 2:** The double logarithmic equations and binding constants of myristic acid (MA) and linoleic acid (LA) with rice protein (RP) at different temperatures were obtained.

Sample	T/K	Double Logarithmic Equation	*K_a_*/L mol^−1^	*n*	*R* ^2^
MA–RP	298	lg[(F_0_ − F)/F] = 0.5345lg[Q] − 0.5265	0.3030	0.5345	0.9831
	310	lg[(F_0_ − F)/F] = 0.4219lg[Q] − 0.6696	0.2140	0.4219	0.9582
LA–RP	298	lg[(F_0_ − F)/F] = 0.4404lg[Q] + 0.3740	1.0899	0.4404	0.9749
	310	lg[(F_0 −_ F)/F] = 0.7884lg[Q] − 0.8540	0.1401	0.7884	0.9702

**Table 3:** Thermodynamic parameters of myristic acid (MA) and linoleic acid (LA) binding to rice protein (RP).

Sample	T/K	Δ*G* (kJ mol^−1^)	Δ*H* (kJ mol^−1^)	Δ*S* (kJ mol^−1·^K^−1^)	*Type of Interaction*
MA–RP	298	−10.04	−7.34	9.06	Electrostatic interaction
	310	−10.15			Electrostatic interaction
LA–RP	298	−163.10	−116.1	157.73	Electrostatic interaction
	310	−165.20			Electrostatic interaction

## Data Availability

The original contributions presented in the study are included in the article, and further inquiries can be directed to the corresponding authors.
